# DO OLDER ADULTS AND THEIR CHILDREN AGREE ABOUT THE USE OF LOCATION TRACKING, IN-HOME SENSORS, AND WEB-CAMERAS?

**DOI:** 10.1093/geroni/igz038.1357

**Published:** 2019-11-08

**Authors:** Clara Berridge

**Affiliations:** University of Washington, Seattle, Washington, United States

## Abstract

On the dyad and aggregate level, we compare preferences of older adult women and their adult children for three remote monitoring technologies: location tracking, in-home sensors, and Web-cameras. Their assessments of each technology’s impact on privacy, safety, independence, freedom, relationship with family member, social life, and identity are also compared. Twenty-eight individual, in-depth structured interviews were conducted with 18 women who are Meals on Wheels clients and 10 of their adult children. Adult children preferred each technology more than their mothers did and underestimated both their mothers’ ability to comprehend the functions of the technologies and the importance of engaging them fully in decision making. For both groups, privacy was the most-cited concern, and participants perceived significant overlap between values of privacy, independence, identity, and freedom. Shared decision-making tools are needed to promote remote monitoring use consistent with older adults’ values and to prevent conflict and caregiver overreach.

